# What to Eat

**Published:** 2007-03-15

**Authors:** Camille Martin

**Affiliations:** Northrop Grumman Information Technology, Centers for Disease Control and Prevention, Atlanta, Ga

**Figure:**
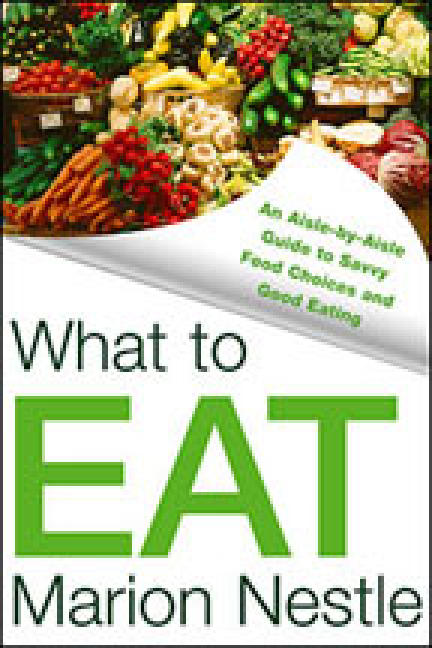


More than a book about choosing healthy foods, *What to Eat* explains how the conflict between business goals and consumer interests creates public confusion about nutrition. Marion Nestle, a respected nutrition professor from New York University, demonstrates how commercial, industrial, and political issues determine what constitutes our food supply and provides readers with a revealing look at the standard practices of government agencies, retailers, and food manufacturers that the complex world of food sales comprises. Nestle wrote *What to Eat* with the consumer in mind and hopes the reader will use the information presented to make informed choices when buying food. The book is organized as a supermarket tour, an interesting and helpful format that allows the reader to more easily apply newly acquired knowledge when purchasing foods.

The public is frequently given advice to choose healthy foods, but people have little direction when it comes to navigating the supermarket and actually making these choices. In *What to Eat*, Nestle has readers envision themselves traveling down grocery store aisles, a technique that helps reinforce the information and makes it easier to remember. This format is different from traditional approaches, which use food categories or groups to educate consumers, and it is effective. Nestle additionally takes complicated food-related issues — issues consumers frequently hear about abstractly but have no way to directly relate to foods they buy — and interprets them as they apply to common supermarket products.

Beginning with the produce section, Nestle gives a detailed explanation of the difference between organic and conventionally grown foods. She explains how organic farming is regulated by the government and provides the social, health-related, and environmental implications of buying organic products. Continuing with the dairy and meat sections, Nestle presents the benefits and potential disadvantages of buying soy products and exposes the realities of meat processing and handling. She discusses the problem of methylmercury contamination of fish and gives information about choosing and preparing foods to decrease the chance of contracting foodborne illnesses. In the section on prepackaged foods, Nestle incorporates a comprehensive explanation of the nutrition facts panel to simplify the difficult process of determining what packaged foods are high in fiber and low in sugar. (Her reproduction of an ingredient listing quickly gets the point across, a demonstration that is informative and entertaining). She describes hydrogenation — a commonly used manufacturing technique through which unsaturated fats are converted into *trans* fats — a process that is used to ensure packaged foods do not spoil rapidly. Nestle covers products from bread to baby food, leading readers around her hypothetical store and encouraging them to decide for themselves what foods are worth buying.

However, before consumers can make decisions about what foods are healthy and worth purchasing, they must be aware of the external influences that drive food sales. This is the underlying premise of *What to Eat*. Nestle encourages readers to think like consumers — not simply dieters, concerned parents, or sports enthusiasts — because manufacturers and retailers target customers as potential buyers. Her goal is to make readers aware of the incredible amount of marketing involved in selling food and that clever packaging, product placement, and even supermarket floor plans are strategically designed to influence consumer choices. Nestle discusses in absorbing detail how political involvement (e.g., government subsidies, efforts of lobbying groups) affects food availability. She explains how manufacturers' payment of expensive slotting fees determines the location of foods throughout the supermarket, which ultimately affects consumers' selection of foods. Nestle makes the reader aware that advertising continues inside the supermarket, illustrating that tremendous research and resources go into designing food packages with the ultimate goal of persuading shoppers to buy those foods. She describes tactics used by large manufacturers to sell food, some of which seem questionable at best: using advertising to target children, pressuring government agencies to allow spurious health claims on food packages, and taking legal action to prevent smaller companies from producing healthier products that may outsell theirs.

Throughout the book, Nestle reinforces her point that food is primarily a business; manufacturers are under extreme pressure to increase their profits, and this pressure is what compels them to relentlessly advertise and market the foods they sell. Nestle wants readers to know that if they do not see themselves as informed consumers, they are more likely to be subconsciously persuaded to purchase foods that are cheapest to make, most expensive to buy, and, not coincidentally, the least healthy.

*What to Eat *is lengthy, and some of the information can be cumbersome for readers encountering these concepts for the first time (e.g., intricate descriptions of policy making). However, this is offset by the benefit to consumers of actually having this information at all. Although Nestle occasionally discusses her preferences about foods and her opinion about the issues surrounding them, the information given is unambiguous and unbiased. The facts are there for the reader to examine, and Nestle encourages a personal evaluation and interpretation so that the consumer can choose intelligently and independently. The book has been meticulously researched and referenced, and its format makes it a tremendously useful reference guide. In *What to Eat*, Marion Nestle presents an excellent, revealing account of how foods are produced, marketed, and sold and gives consumers the tools to consider the social, environmental, economic, and health-related implications of purchasing food.

